# Global Quantitative Modeling of Chromatin Factor Interactions

**DOI:** 10.1371/journal.pcbi.1003525

**Published:** 2014-03-27

**Authors:** Jian Zhou, Olga G. Troyanskaya

**Affiliations:** 1 Lewis-Sigler Institute for Integrative Genomics, Princeton University, Princeton, New Jersey, United States of America; 2 Graduate Program in Quantitative and Computational Biology, Princeton University, Princeton, New Jersey, United States of America; 3 Department of Computer Science, Princeton University, Princeton, New Jersey, United States of America; University of Chicago, United States of America

## Abstract

Chromatin is the driver of gene regulation, yet understanding the molecular interactions underlying chromatin factor combinatorial patterns (or the “chromatin codes”) remains a fundamental challenge in chromatin biology. Here we developed a global modeling framework that leverages chromatin profiling data to produce a systems-level view of the macromolecular complex of chromatin. Our model ultilizes maximum entropy modeling with regularization-based structure learning to statistically dissect dependencies between chromatin factors and produce an accurate probability distribution of chromatin code. Our unsupervised quantitative model, trained on genome-wide chromatin profiles of 73 histone marks and chromatin proteins from modENCODE, enabled making various data-driven inferences about chromatin profiles and interactions. We provided a highly accurate predictor of chromatin factor pairwise interactions validated by known experimental evidence, and for the first time enabled higher-order interaction prediction. Our predictions can thus help guide future experimental studies. The model can also serve as an inference engine for predicting unknown chromatin profiles — we demonstrated that with this approach we can leverage data from well-characterized cell types to help understand less-studied cell type or conditions.

## Author Summary

Chromatin, like many other molecular biological systems, is composed of multiple interacting factors. Our knowledge about chromatin factors is mostly qualitative, and such qualitative knowledge can be insufficient for predicting collective behaviors. It's also extremely challenging to study collective behaviors involving multiple interacting factors through genetic and biochemical experiments. An alternative approach is to leverage large-scale genome-wide chromatin profiles and statistical modeling to create predictive models and infer underlying interaction mechanisms based on these observed high-throughput data. In this study, we developed a novel maximum entropy-based modeling approach to quantitatively capture interactions between chromatin factors at the same genomic location, which we see as a step toward quantitative understanding of chromatin organization that involves a system of multiple interacting factors. We applied this quantitative model to successfully infer functional properties of chromatin including interactions between chromatin factors. Furthermore, the model predicts unmeasured chromatin profiles with high accuracy based on its inferred dependencies with other factors within and across cell-types. Thus our modeling approach effectively ultilizes large-scale chromatin profiles to dissect chromatin factor interactions and to make data-driven inferences about chromatin regulation.

## Introduction

Genome-wide large-scale chromatin profiling projects such as modENCODE/ENCODE [1], [2] have provided unprecedented measurements of chromatin factor combinatorial patterns, enabling a holistic view for understanding chromatin. Chromatin factors, including histone-modifications and non-histone chromatin proteins, are direct contributors to the diverse repertoire of chromatin regulation to gene expression. Although we have gained much knowledge on functions of individual chromatin factors, understanding the collective code of chromatin factor patterns and their underlying mechanism has remained a key challenge. Understanding the collective behavior and function of chromatin factors is challenging as the establishment and maintenance of chromatin factor patterns are usually orchestrated by complex molecular events involving multiple chromatin components, and chromatin factors can affect the functional interpretation of each other [3].

Despite the increasing availability of large-scale chromatin data, no model has been capable of quantitatively explaining frequencies of observed multi-dimensional chromatin factor patterns, answering the important questions such as: Can we build a predictive model of collective chromatin factor pattern frequencies based on the interactions between chromatin factors? And can we deduce the interaction strengths that best explain the observed chromatin factor patterns? Such a quantitative model would have a clear criterion for validation (accuracy in predicting pattern frequencies), would give interpretable estimation of quantitative dependency strengths between chromatin factors, and could provide quantitative inference of unmeasured chromatin factor profile based on partial data. To date, no such quantitative model has been established for chromatin factor patterns and interactions. Some previous studies have focused on inferring the conditional dependency structure between chromatin factors through Bayesian network structure learning [4]–[6], but Bayesian network models rely on strong assumptions of directed and acyclic dependency structure. While some interactions can be directed and better modeled by Bayesian network, this assumption will not hold in general as many physical binding interactions between chromatin factors are undirected. In addition, these studies have been limited to inferring an ensemble of candidate dependency structures with heuristic search, and have not been able to establish a parameterized probabilistic model of chromatin factor patterns based on dependencies among chromatin factors. A recent study proposed sparse partial correlation for detecting interactions between histone modifications [7]. Partial correlation matrix represents conditional independency structure between variables when the distribution is multivariate Gaussian. However, the distributions of ChIP-chip chromatin factor binding signals highly deviate from multivariate Gaussian distribution (Figure S4, Text S1). Probabilistic models including hidden Markov model and dynamic Bayesian network methods have been applied to chromatin data to segment the genome into functional states using probabilistic models, and these models have offered insights into association between chromatin states and function [8]–[11]. But these models either ignore dependencies between chromatin factors or use a highly simplified model of such dependency.

**Figure 1:**
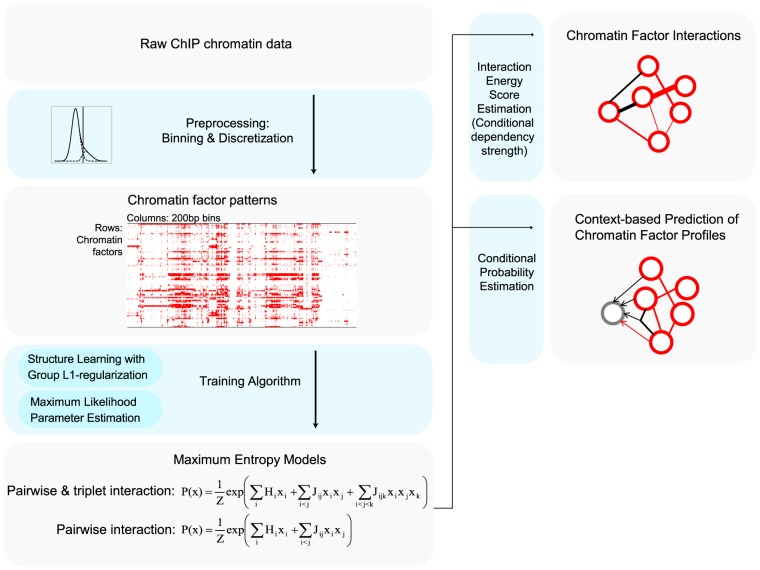
Schematic overview of chromatin factor interaction maximum entropy model. Chromatin factor patterns were extracted from ChIP data by binning and thresholding algorithms (Methods). We then learned a maximum entropy model that estimates the distribution of chromatin factor patterns by low-order (pairwise or pairwise & triplet) interactions. The model was then applied to prediction of chromatin factor interactions and performing context-based prediction of chromatin profiles.

In order to improve quantification of chromatin-factor interactions and establish a probabilistic model that enables data-driven inference of chromatin factor profiles, here we developed a quantitative maximum-entropy-based framework for global integrative chromatin profile analysis that is capable of learning and leveraging interaction strengths between chromatin factors from large-scale chromatin data (Figure 1). We utilized this quantitative model to provide new insights into chromatin function on a range of scales. Our approach simultaneously estimates both the dependency strengths between chromatin factors and the chromatin ‘codebook’ – frequency distribution of each chromatin factor combinatorial binding pattern, or chromatin code, observed at each bin of genomic location. We found that due to the nature of high dimensionality of chromatin code and enormous number of possible combinatorial patterns, modeling the pattern distribution requires capturing the correlation structure with compact representation of dependency, and we achieved this by explaining the correlation structure by estimated pairwise and triplet interactions between chromatin factors. Our maximum entropy approach is based on the chromatin profiles for *Drosophila melanogaster* S2-DRSC cell produced by the modENCODE project for estimating statistical dependency strengths between 73 chromatin factors. Our approach is, to our knowledge, the first that is capable of capturing higher-order chromatin factor interactions through group L1-regularization-based structure learning, which improves parameter estimation in high dimensional space. The resulting model accurately reproduced the observed chromatin code frequencies, and the dependency strengths of chromatin factors estimated by the model accurately predicted experimentally determined interactions. Furthermore, the interaction model can serve as a context-based chromatin factor profile inference engine. Given data for a subset of chromatin factors, the model can provide accurate inference for chromatin factor profiles based on interaction information and the known profiles. Interestingly, comparable estimation accuracy can be achieved for most chromatin factors even when using the model to make predictions for a different cell type, the BG3 cell, suggesting the potential for leveraging data from highly characterized cell type to help understand less-characterized cell types.

## Results

To quantitatively model not just the structure, but also strength of interactions among chromatin factors, including higher-order relationships, we developed a maximum-entropy-based chromatin code modeling framework. Using this framework, we constructed the quantitative model of chromatin factor patterns for Drosophila S2-DRSC cell in normal culture condition. This dataset, produced by the modENCODE project, is one of the most extensively profiled chromatin datasets with a wide coverage of 73 non-histone chromatin proteins, histone modifications, and histone variants/subunits. Our model can predict both strength and sign (positive versus negative) of interactions between chromatin factors incorporating both pair-wise and higher-order interactions, and can accurately predict experimentally determined chromatin factor relationships. The strengths of interactions captured by the model enable us to quantitatively describe crosstalk among chromatin factors. Furthermore, the quantitative model allows inference of unmeasured chromatin profiles based on partial measurements, even in different cell types and conditions, and thus can help understand chromatin in diverse less explored cell types or conditions.

### A quantitative maximum-entropy model of chromatin factor interactions

Maximum Entropy modeling provides a flexible framework that allows building models that discount indirect or transitive relationships, with minimal assumptions. Intuitively, maximum entropy modeling works by choosing the most uniform/least structured, or maximum entropy, probability distribution while ensuring consistency with a chosen set of observed statistics. For modeling chromatin codes, natural choices for these statistics are frequencies of individual chromatin factors and pairs of factors, both of which can be reliably measured (Methods). On the technical level, the model can be viewed as determining the probability of observing a multivariate pattern by exponential of the “energy function” of the pattern, such as
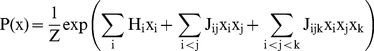
(1)


where 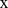 represents a chromatin factor pattern by a binary vector representing chromatin factors observed at a genomic location, and energy parameters 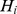, 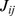
and 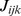 tune the occurrence frequency of single factors, interaction strength between two factors, and triplet interaction strength respectively. 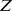 represents the normalization factor or partition function that ensures that the sum of probability of all possible patterns equals 1. The energy of a chromatin factor pattern is the sum of the self-energies of all presented chromatin factor and interaction energies of all existing interactions. Maximum entropy modeling has been successfully used to learn pair-wise interaction models for a wide range of other complex biological systems, including modeling correlation structure of neuron firings from multiple neurons[12], capturing physical interactions between amino acids in protein sequences through identifying direct evolutionary coupling [13]–[15], and modeling co-evolved mutation pattern in virus protein [16].

An additional advantage of our modeling framework is that it can be readily extended to prediction of higher-order interactions. Many complex interactions cannot be accurately captured by pair-wise interactions, such as cooperative multivalent binding or inhibition of binding between histone mark and reader protein by other marks [3], [17]. Such higher-order relationships are very challenging to study without specific hypotheses to guide experiments. To allow the maximum entropy framework to incorporate higher-order interactions without overfitting, we extended our framework to introduce L1-regularization to enable accurate estimation of the free parameters by selecting for an optimized subset of non-zero interactions. More specially, we applied a group L1-regularization method to select for structure of the model [18], and then estimated the model parameters with maximum likelihood to get an unbiased maximum entropy model.

**Figure 2:**
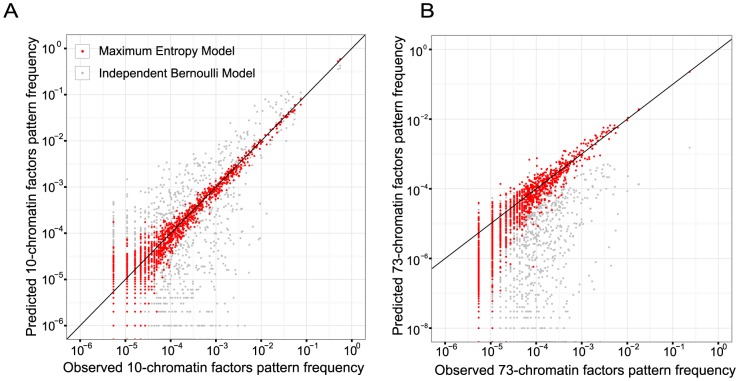
Maximum entropy model accurately predicts high-order chromatin factor pattern frequencies in the data. For evaluation we predicted frequencies of chromatin factor combinatorial binding patterns each involving either randomly selected 10 chromatin factors (A) or all 73 chromatin factors (B) from model, and compared against observed frequencies in test set data. Red dots represent estimations from maximum entropy model with up to 3^rd^ order interactions learned with regularization and fine-tuning; gray dots show estimations from independent Bernoulli model. Independent Bernoulli model assumes independence between occurrences of different chromatin factors. The diagonal line is the identity line.

**Figure 3:**
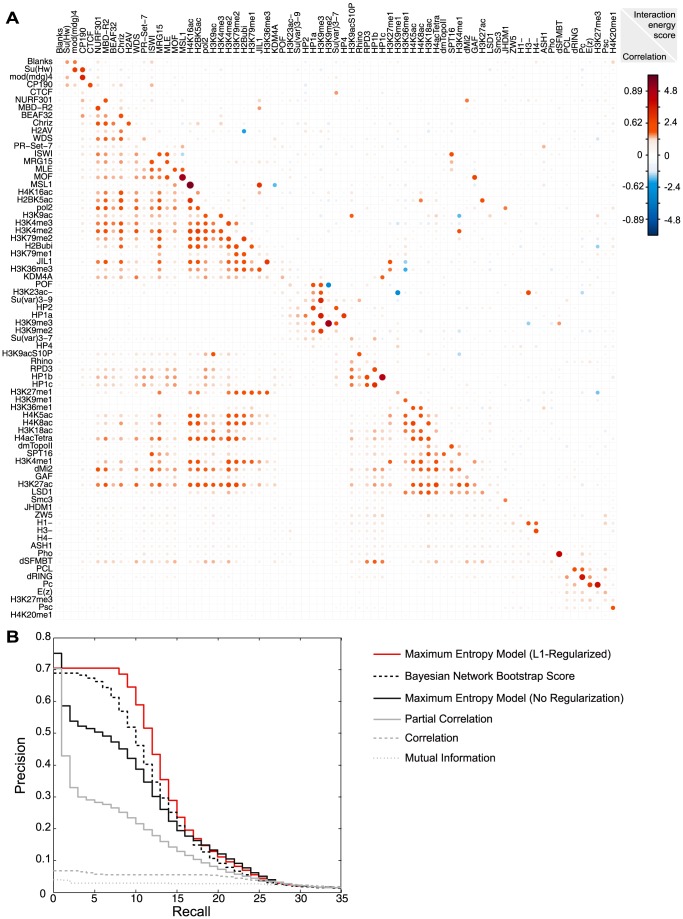
Model provided accurate predictor for experimentally validated chromatin factor interactions. (A) Heatmap visualization of maximum entropy model pair-wise interaction energy scores (upper right) compared with correlation z-scores (lower left); The heatmap is ordered to position positive interactions close to diagonal so positively interacting factors tend to be adjacent to each other. H3K23ac-, H1-, H3-, H4- represent the depletion of these factors respectively. For comparison with interaction scores, correlations were transformed to z-scores by Fisher transformation and rescaled to make standard deviation equal to the standard deviation of pair-wise interaction energy scores. In figure legend, the corresponding correlation (left) and interaction energy score (right) at each z-score level is shown. The interaction energy score prediction is robust to changing bin size in data processing (Figure S2). (B) Precision-recall curves for predicting known interactions. Precision-recall curve shows the performance of using interaction energy score to classify interaction at all thresholds. Precision-recall curve of L1-regularized pair-wise interaction maximum entropy model interaction energy scores (red, solid) is compared to unregularized pair-wise interaction maximum entropy model interaction energy scores (black, solid), Bayesian network bootstrap score (black, dashed), Pearson correlation coefficients (grey, dashed), Partial correlation (grey, solid), and mutual information (grey, dot). Maximum entropy models, Bayesian network model and mutual information are computed on discretized data, while correlation and partial correlation were computed on continuous data without discretization.

We evaluated the model performance by calculating coherence score of the model and comparing predictions of multi-chromatin factor pattern frequencies by the model with observed frequencies (Figure 2). Both evaluations were calculated on hold-out test set data, which were withheld from all training procedures. Coherence scores were measured by exponential of mean log-likelihood of each observed pattern in the test data given the model. We trained and tested performances of several maximum entropy models, and normalized the coherence score to the best model score (Table 1). Maximum entropy models including up to 3^rd^-order interaction with regularization significantly improved over pair-wise maximum entropy models with or without regularization. Maximum entropy model incorporating sparse 3^rd^ order interactions trained with group L1-regularization and maximum likelihood fine-tuning gave the best performance, and it demonstrated remarkable consistency with observed multi-chromatin factor pattern frequencies, even though only low-order statistics (chromatin factor frequencies and frequencies of pairs / triplets of factors) were used for parameter estimation (Figure 2). As most chromatin profiles are clearly highly correlated (Figure 3A), it is not surprising that the multivariate Bernoulli distribution model assuming independence between chromatin factors gave very low coherence score. Therefore, we decided to use maximum entropy model with 3^rd^ order interactions as our best probabilistic model for chromatin factor patterns. In this model, 7.6% of pairwise interaction energies and 71.9% of triplet interaction energies are set to zero by L1-regularization. To our knowledge, this model is the first quantitative model of chromatin factor pattern frequencies considering chromatin factor interactions and the first one capable of predicting higher-order interactions.

**Table 1:** Comparison of different models on hold-out data.

Model	Coherence score (Normalized)	Number of Parameters
3^rd^-order interaction model (Regularization+Fine-tuning)	1.00	19975 (73+2429+17473)
3^rd^-order interaction model (Regularization)	0.97	19975 (73+2429+17473)
Pairwise interaction model (No regularization)	0.65	2701 (73+2628)
Pairwise interaction model (Regularization+Fine-tuning)	0.63	2512 (73+2439)
Pairwise interaction model (Regularization)	0.51	2512 (73+2439)
Independent Bernoulli model	0.0001	73

Coherence score was calculated as exponential of the mean log-likelihood of each chromatin code in the hold-out test data, and was normalized by the best model coherence score (hence the 3^rd^ order interaction model with regularization and fine tuning has normalized coherence score of 1). Numbers of non-zero parameters for self-energy, pairwise interaction and third-order interactions are shown in parenthesis, in that order.

### Maximum entropy model accurately predicts known chromatin factor interactions

**Figure 4:**
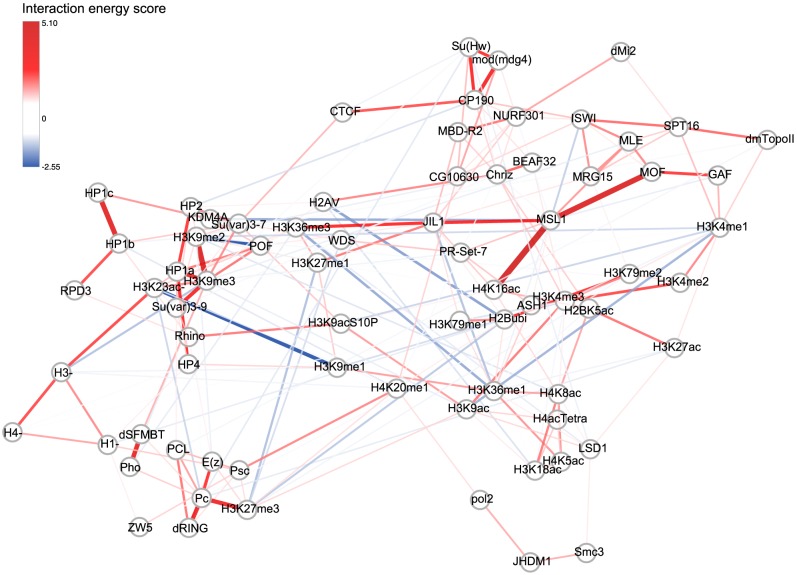
Pair-wise interaction network organization structure of chromatin factors. Each node represents a chromatin factor and each edge represents a pair-wise interaction. Edge color indicates sign and strength of interaction energy score (red indicates positive interaction while blue indicates negative interaction). Only interactions with interaction energy score 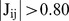 are shown.

Interactions between chromatin factors are critical for organization and regulatory function of chromatin, and are challenging for large-scale experimental analysis, yet our chromatin model provides de novo estimation for chromatin factor interactions. The model can capture interactions between chromatin factors quantitatively through their tendency to co-occur or not to co-occur in chromatin profiles, including interactions resulting from direct physical interaction, enzyme-substrate relationship, or co-binding to the same DNA sequence or unidentified factors. Specifically, for pair-wise interactions, we used the interaction energy score 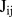 from regularized pair-wise interaction model (log fold change in pattern frequency attributed to this interaction) as the metric for interactions strength (Table 2, Table S1, Figure 3, 4). Importantly, interaction energy score in the maximum entropy model estimates interaction between two factors conditioned on all other factors in the model, meaning that it discounts indirect or ‘transitive’ correlations that are measured by pair-wise statistics such as correlation (Figure 3A, S1B). The property of dissecting out indirect interactions is critical to understanding the cross-talk among chromatin factors, as otherwise indirect correlations prevent the identification of individual direct interactions by indiscriminately connecting large groups of chromatin factors (Figure 3A). Note that increasing the coverage of interacting factors in the data enables our model to dissect more direct versus indirect interactions, thus the model benefits from the large-scale collaborative efforts in chromatin profiling like modENCODE and ENCODE projects [1], [19], and is expected to improve further as the coverage of chromatin factors in such projects grows.

**Table 2:** Top 20 predicted positive pairwise interactions based on pairwise interaction model with regularization.

Chromatin factors		Interaction energy score
H4K16ac	MSL1	5.10
**MOF**	**MSL1**	4.56
H3K9me2	H3K9me3	4.53
HP1b	HP1c	4.21
**dSFMBT**	**Pho**	3.76
**H3K27me3**	**Pc**	3.52
**dRING**	**Pc**	3.50
**H3K9me3**	**HP1a**	3.21
**CP190**	**mod(mdg4)**	3.10
**H3K9me3**	**Su(var)3–9**	3.05
JIL1	MSL1	2.64
**CP190**	**Su(Hw)**	2.64
**HP1a**	**HP4**	2.58
H3K36me3	JIL1	2.56
**mod(mdg4)**	**Su(Hw)**	2.49
H2Bubi	H3K79me2	2.45
E(z)	Pc	2.43
HP1a	HP2	2.42
GAF	MOF	2.40
H3K23ac-	H3-	2.29

Bold pairs are in evaluation standards (experimentally validated).

Many strong interactions identified by our model have been verified by experiments in previous studies. Among the top ten predicted interactions, several are direct physical interactions between proteins that are part of larger complexes (Table 2), including the dosage-compensation complex (DCC) components MOF - MSL1 [20], PhoRC complex components Pho – dSFMBT [21], Polycomb-repressed chromatin mark and binding protein Pc - H3K27me3 [22], heterochromatin mark and binding protein H3K9me3 - HP1 [23], gypsy insulator complex components CP190 - mod(mdg4) [24], and heterochromatin mark and writer H3K9me3 - Su(var)3-9 [25]. As expected, indirect interactions mediated by factors not included in the model can also be captured: for example MSL1 and MLE were estimated to positively interact, and their interaction with each other is likely indirect and mediated by roX RNAs[26], which of course are not part of this dataset. Communities of histone marks and chromatin proteins connected by positive interactions are also consistent with current knowledge (Figure 4).

The model has been shown to be a precise probability model of hold-out data with high coherence score, but a separate evaluation is required for benchmarking the model's ability to identify direct interactions, which also depends on the coverage of chromatin factors included in the dataset. To quantitatively evaluate performance of interaction energy scores in rediscovering known interactions, we collected a set of experimentally supported positive interactions including physical interaction, histone-modification – chromatin protein binding, and enzyme-substrate interaction based on public curated databases and our manual curation (Table S2). L1-regularized pair-wise interaction Maximum entropy model demonstrated a remarkably high precision in capturing known interactions, with 10/15 top predictions being experimentally verified interactions (46 fold of background precision of 1.45%), suggesting its potential in predicting unknown interactions (Figure 3B). This performance far surpassed common ‘local’ pair-wise measures including correlation and mutual information, which have below 10% precision using any threshold (Figure 3B). Partial correlation gives better performance than correlation but the performance is much lower than the maximum entropy model (Figure 3B), and this is likely because the distribution of chromatin factor pattern is not multivariate Gaussian, violating the assumptions required for interpreting partial correlation as conditional independence structure (Figure S4, Text S1). We also applied the Bayesian network bootstrap score approach [4], [5], and it underperforms the L1-regularized maximum entropy model (Figure 3B). It is further important to emphasize that in contrast to our maximum entropy model, the strength and sign of interaction are not explicitly learned by the Bayesian network method (Figure S1A).

Given the high performance on hold-out data and the high enrichment of known interactions among the top predicted interacting pairs, our model is promising for predicting novel interactions (Tables 2, S1). Analysis of top predictions identified additional experimentally supported interactions not included in our standards, as well as promising novel predictions. Other than the interaction between MSL1 and MLE mentioned above, CP190-CTCF was also reported to be co-immunoprecipitated [27]. Another interesting prediction is the GAF-MOF positive interaction. A few lines of evidence support potential association between them: The GAF binding motif is enriched in poised MSL complex entry sites, and MOF is a component of MSL complex [28]. Moreover, GAF has been shown to affect at least one specific MSL complex entry site on X-chromosome [29]. Some other predictions are supported by motif or domain in protein sequences. For example, HP1b-RPD3 is predicted to be a positive interaction, and shadow domain of HP1b is predicted to bind PXVXL motif [30] while we found RPD3 has a very close PXVXI sequence motif supporting potential interactions. Several novel predictions show potential connection between different chromatin-based processes, for example, histone chaperone SPT16 is predicted to strongly interact with topoisomerase II. SPT16 and topoisomerase II have been co-purified in a complex in human HeLa cell line[31], and SPT16 is a component of FACT complex which is involved in chromatin remodeling during elongation. This indicates that chromatin remodeling complex and DNA topoisomerase may function together in coordinating chromatin structure.

#### Maximum entropy model predicts higher-order interactions

Higher-order interactions involving more than two chromatin components have not been well studied except for a few examples [3], [17]. This limits a systematic evaluation of these higher-order predictions, although our finding that third order interactions improved maximum entropy model coherence score performance likely indicates third order interactions well captured chromatin factor cross-talk represented in the data. These triplet interactions were estimated from group L1-regularized 3^rd^ order interaction models and capture effects not explained by pair-wise interactions (Table S3). Positive or negative triplet interactions indicate positive or negative cooperatively of co-occurrence. Experiments on synthetic dataset have shown that this group L1-regularization algorithm is capable of recovering true high-order interactions [18]. An interesting example of the predicted higher-order interactions is the negative triplet interaction among CP190, CTCF, and Su(HW) (Table S3). CP190 and Su(Hw) are known to interact and both showed co-localization with CTCF [32], and indeed our model captured strong positive pair-wise interactions CP190-CTCF and CP190-Su(Hw). We hypothesize that this negative triplet interaction indicates direct or indirect ‘competition’ between CTCF and Su(Hw) over co-binding with CP190 – binding of one protein may ‘suppress’ the binding of the other to CP190. This observation supports a model where CP190 forms different insulator complexes with different groups of proteins, possibly directed by different DNA sequence motifs. Our predicted higher-order interactions provide hypotheses that can guide future experimental studies in this complex and large search space.

### Maximum entropy model enables accurate chromatin profile inference within and across cell types

Strong interactions estimated among chromatin factors suggest that we may use profile information from a subset of chromatin factors to infer other profiles based on the model. As a generative probabilistic model, the chromatin model can in principle give probability of occurrence of any chromatin factor(s) conditioned on the context of known binding profile of other chromatin factors. Furthermore, here we investigate whether such inference can be performed across cell types.

#### Prediction of chromatin profile based on partial chromatin data

**Figure 5:**
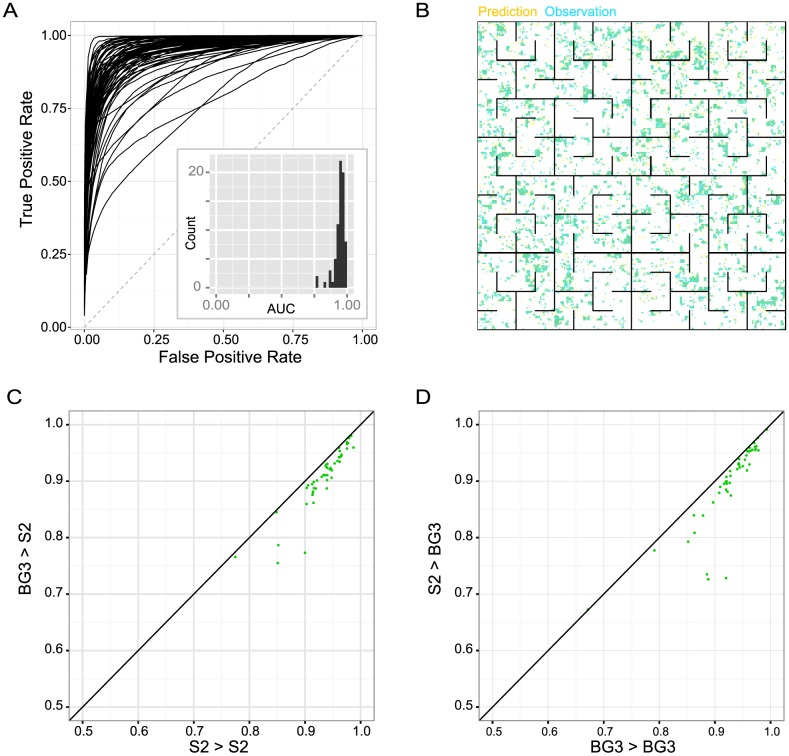
Context-based intra- and inter-cell type chromatin factor profile predictions achieve high overall performance. (A) Prediction performances on hold-out chromatin factor profiles based on partial data and chromatin model. Chromatin factor profile predictions are compared with observed chromatin profiles using receiver operating characteristics (ROC) that shows true positive rate (y-axis) and false positive rate (x-axis) at full range of prediction thresholds. The diagonal line (dashed) shows expected performance of random classifier. The histogram shows frequency distribution of area under ROC curves (AUC). (B) Comparison of predicted and observed S2 cell H3K18ac chromatin profile. ChIP profile is visualized as the space-filling Hilbert curve as in [11], therefore adjacent genomic locations are also close to each other in this 2D representation. Predicted profile based on BG3 cell model is colored yellow, with darker color showing higher probability; Observed binarized profile is colored blue; Overlap between predicted and observed profile is therefore green. H3K18ac is an example chromatin factor which cannot be accurately inferred from any other single chromatin profile (the highest correlation coefficient with H3K18ac is 0.37). (C, D) Comparison of inter-cell type versus intra-cell type chromatin profile prediction performances. Performance is measured by AUC. ‘->’ indicates which cell lines the model is trained for and tested on, e.g. S2->BG3 represents predicting BG3 cell data with model trained on S2 data.

We first evaluated the predictability of chromatin profiles. Applying the 3^rd^ order maximum entropy model to the hold-out test set data, we predicted each chromatin factor profile from the remaining profiles and evaluated the prediction with measured data. Most chromatin factor profiles can be well predicted given the model and the rest chromatin profiles. 66/73 (90%) chromatin factor profiles were predicted with an AUC (area under the Receiver Operating Curve) larger than 0.90 (Figure 5A, Table S4). AUC can be interpreted as the probability that the prediction score of a randomly chosen positive genomic bin is larger than the prediction score of a randomly chosen negative genomic bin.

#### Chromatin profile predictions across cell types

Most current large-scale epigenome mapping projects take the strategy of characterizing a large number of chromatin factors for only a few cell types while characterizing a much smaller subset for other cell types [2], [33], and it is costly to perform extensive chromatin profiling in new cell types or under new conditions. Therefore, we investigated whether it is possible to leverage the dependency information captured in the model trained on the data from a relatively comprehensively profiled cell line to help predict unmeasured chromatin profiles in less-characterized cell lines.

Conservation of chromatin factor interactions is critical for the feasibility of cross cell type prediction. Therefore we learned regularized pairwise interaction maximum entropy models for each of S2 cell and BG3 cell on a set of 47 chromatin factors shared between datasets of the two cell lines, and then compared the resulting models. Comparison of the two models shows high correlation of interactions energy scores (0.77) (Figure S3), despite relatively low correlation between chromatin profiles for the same chromatin factor in the two cell types (mean = 0.43, standard deviation = 0.17). Therefore it appears promising to attempt predicting chromatin profiles across cell types based on conditional probability estimators.

To demonstrate feasibility of cross cell type prediction, we trained 3^rd^-order maximum entropy models using data from either S2 cell or BG3 cell separately, and then we tested each model on predicting test set chromatin profile from both the same and the other cell type. The results showed that chromatin profile prediction using the model trained on data of another cell type achieves similar performance as the model trained with data from the same cell type (2.8% decrease predicting BG3 cell profile, 3.2% decrease for predicting S2 cell profile; Figure 5C–D). An example of such prediction is shown in Figure 5B. These results confirmed the potential of leveraging our models to estimate unmeasured chromatin profiles in less-characterized cell types, enabling improved understanding to chromatin in diverse cell-types and conditions and planning of future chromatin profiling experiments by prioritizing chromatin factors potentially involved in the research question of interest based on the model predictions or chromatin factors that are less-predictable from known chromatin profiles.

## Discussion

We developed a global modeling framework for chromatin profiles based on a generative model of chromatin codes that, to our knowledge, is the first probabilistic model that captures both pairwise and higher-order interactions among chromatin factors. Applying this method to the large-scale chromatin profiles, we get a high accuracy, data-driven estimator of interactions that underlie the formation of chromatin factor patterns. Moreover, our methods provided effective tools to make data-driven prediction of unknown chromatin profiles based on context, and showed feasibility of prediction across cell types.

**Figure 6:**
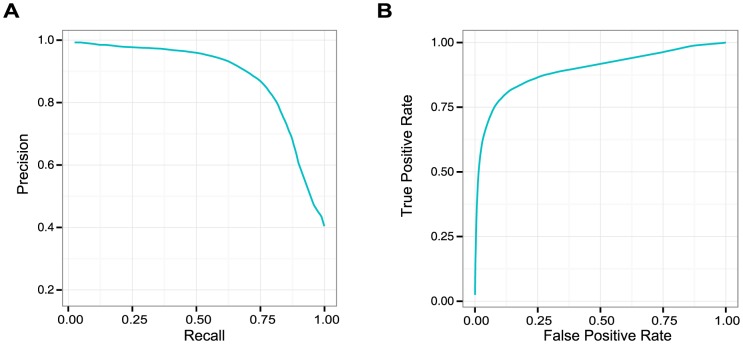
Extended chromatin model well predicts actively transcribed genomic regions. Predictions are compared with precision-recall curve (A) and receiver-operating characteristics (ROC) curve (B) at full range of prediction thresholds.

Our model is also extendable to include more information or interaction types. To demonstrate this, we applied the model to predict actively transcribed genomic regions. We discretized transcription level to high and low classes by fitting a mixture model [34], and included transcription level at each genomic bin in chromatin factor patterns for learning the maximum entropy model. Prediction for transcription was then performed by conditional probability estimation given all other chromatin factor profiles. While previous research has demonstrated high predictability of gene expression from chromatin profiles [1], [35], [36], here we showed that transcription can be accurately predicted based on the chromatin profiles even without any prior knowledge about gene structure in test data (Figure 6), and as a generative model this model allows more diverse types of inference than simply predicting expression, such as inferring chromatin marks profiles while conditioning on expression level. The model can also be potentially extended to model both discrete and continuous variables [37].

To fully leverage the power of large-scale chromatin profiles, using a global statistical model like the maximum entropy model allows one to focus on direct interactions even when transitive and indirect interaction signals are prevalent in chromatin profiling data. However, it is important to note that such models will detect interactions that are indirect when the factor that mediates this indirect interaction is not included in the model. Furthermore, maximum entropy modeling is especially suited for many biological systems as it makes minimal assumptions. This is important in this problem because we have little prior knowledge about quantitative nature of interaction. Over-parameterization can be a limitation for models involving too many potential interactions, which is common when a model includes a large number of factors or when higher order interactions are considered as in our case. We demonstrated that regularization helps learning better biological interactions from data and selecting features for building higher-order models.

Histone modifications are known to facilitate or repress each other through crosstalk [3], and our modeling approach provides a systematic way to analyze crosstalk patterns. Interestingly, we found that interactions between di-methylation and tri-methylation marks are often positive, for example interactions of H3K9me2 and H3K9me3 or of H3K4me2 and H3K4me3; while mono-methylation and tri-methylation, such as H3K36me1 and H3K36me3 or H3K4me1 and H3K4me3 often have negative interactions. Known examples of histone crosstalk were also captured by the model. For example, H2Bub is reported to stimulate methylation of H3K79me by Dot1L [38]. While Dot1L is not included in the dataset, H2Bub is expected to be positively interacting with H3K79me. Indeed, H2Bub-H3K79me1/2 was predicted to be positively interacting (Figure 3A).

Increase of chromatin profiling capability is expected to provide us access to larger sets of multi-factor chromatin profiles and consequently more powerful models. Our modeling framework is readily applicable to those large-scale chromatin datasets, and it is flexible to be extended it to include different types of features. Although currently only a few cell types have been extensively profiled, we envision that our modeling framework will be widely applicable to new large-scale chromatin data, and our models can also help generate hypothesis and drive experiments in less characterized conditions or cell types by transferring dependencies learned from well-characterized cell types. We are optimistic that as more genome-scale chromatin profiling data become available, quantitative modeling of chromatin organization will enable understanding and modeling how chromatin works in different contexts as a system of multiple interacting factors.

## Methods

### Maximum entropy model formulation

The principle of maximum entropy allows us to construct data-based models with minimal assumptions [39]. While constraining the model to be consistent with observation data on expectation of feature values, the principle of maximum entropy suggests that the optimal distribution is the one with the maximum entropy, or smallest Kullback-Leibler divergence from uniform distribution. Applying the principle of maximum entropy with constraints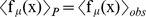 using Lagrange multiplier, where x is the quantity to be modeled and 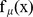 is a feature of x, we get a model with the exponential family distributions form:
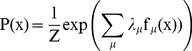
(2)



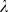
s are free parameters to be estimated from data.

The formulation of model is determined by choice of constraints. For the chromatin model, if we set the features that we expect model to match to be occurrence frequency of each chromatin factor and co-occurrence frequency of each pairs of chromatin factors that we can reliably measure. Thus the model can be written as
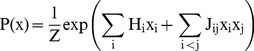
(3)


If we also add triplet frequency constraints, the model becomes equation(1).

Self-energy and pairwise interaction energy parameters 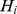 and 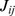 tune the occurrence frequency of single factor and interaction strength between two factors respectively, and triplet interaction energy parameter 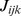 tunes triplet interaction strength. Note that the number of parameters increases exponentially for high-order interactions, if we consider all possible interactions. 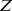 represents the normalization factor or partition function in physics which ensures that the sum of probability of all possible patterns equals 1.

A nice feature of maximum entropy model is that the globally optimal estimation can be uniquely determined since the entropy function is concave, which is not true for Bayesian network for which a complex search space of model structure has to be explored with heuristic methods. It can be shown that the maximum likelihood estimator of parameters in maximum entropy model gives the same unique solution as constrained maximization of entropy. Therefore we can learn the model by an unconstrained optimization for likelihood.

### Model learning

For learning the model, we first performed structure learning using group L1-regularization. L1-Regularization shrinks parameters weakly supported by data to zero. Therefore it reduces the parameter space and allows estimating higher-order interactions. Moreover, we constrained the model structure to be hierarchical: a triplet interaction involving i, j, k will only be non-zero when all three pair-wise interactions i-j, j-k and i-k are non-zero, using algorithms described in [18]. To describe briefly, the model learning objective is written as:
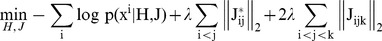
(4)



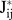
 represents the group of parameters including pair-wise interaction 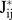
and all third-order interaction parameters that involves both i and j. These constraints enforce hierarchical structure of the model [18]. To speed up computation for structure learning, we replaced likelihood with pseudo-likelihood approximation [18], [40], [41]. Pseudo-likelihood is defined as product of all conditional probability of single variable given the others:
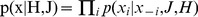
(5)


The negative log pseudo-likelihood is also a convex objective function but it can be exactly optimized much more efficiently. We selected the regularization parameter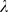 that gives the lowest pseudo-likelihood on validation data.

After structure learning, the non-zero parameters were chosen to be included in the final model and we initialized the model parameters with pseudo-likelihood-based estimation. We found parameters estimated in structure learning step to be sufficient for predicting chromatin factor interactions, but the corresponding probabilistic distribution is not well calibrated. We thus fine-tuned the parameters via the maximum likelihood estimation algorithm. Gradient of model likelihood with respect to 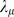 is 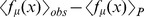. Exact computation of 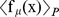 is intractable when number of factors is much larger than 20 as it requires enumerating all possible configurations of X, but it can be well approximated by Monte Carlo Markov Chain (MCMC) sampling. Thus we performed Gibbs sampling to estimate the gradient and performed fixed step-size gradient descent update; the progression of fitting was monitored by Pearson correlation between observed and model-predicted statistics (i.e.
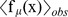
and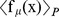). Our implementation of learning procedures utilized Mark Schmidt's Undirected Graphical Model toolbox [42] and code for hierarchical log-linear model [18], with modifications to speed up computation for binary state models we are using.

For computation of chromatin factor pair-wise interaction energy scores, we trained a pseudo-likelihood based L1-regularized 2^nd^-order maximum entropy model. A 3^rd^ –order maximum entropy model trained by structure learning and fine tuning steps were used as our best probabilistic model for chromatin codes and used for estimating 3^rd^ order interactions.

### Validation of chromatin factor interaction estimation

We curated experimentally supported physical interactions and enzyme-substrate interactions between chromatin proteins from BioGRID protein-protein interaction database [43] and literatures. Only interaction evidences from small-scale studies were used. Evaluation standards curation was independent from interaction prediction results. An interaction was included in the evaluation standard if an experimental assay supports direct physical interaction or enzymatic interaction. To stabilize the estimation of precision recall curve, we performed resampling with replacement from evaluation standards for 2000 rounds. Precision recall curves were calculated for each resampled standard, and then all resampled precisions at each recall level were averaged.

### Data sets and processing

Chromatin binding profiles of histones and chromatin proteins were measured by the modENCODE project [1]. ChIP-tilling array probe signals were first standardized using Model-based Analysis of Tilling-array (MAT) algorithm [44], [45]. Probes that are mapped more than once within 1 kb were removed before any analysis. Probe t-values from multiple experimental replicates were averaged, and input control experiments mean t-values (in log scale) were subtracted from ChIP experiments mean t-values to calculate standardized probe signal. Standardized probe signals were binned by 200 bp windows and averaged within each bin. Bins with lower probe coverage than 50% were withheld from further analysis.

After inspecting the distribution of data and applied statistical test for multivariate normality of data (Text S1), we decided it is inappropriate to take the multivariate Gaussian assumption, as enrichment or depletion signal only appear to locate at one tail of the distribution. Instead we applied a signal extraction algorithm to decide statistically optimal decision threshold of signal and noise and discretized the data. We fit a nonparametric mixture model to estimate background distribution and signal distribution assuming only symmetry of the background distribution and that the signal distribution lie on one side of background distribution peak while having negligible probability density on the other side (Figure S4). Enrichment in ChIP experiments were detected for most chromatin factors, except for H1, H3, H4, and H3K23ac for which depletions were detected. We determined optimal decision threshold as the bin signal value at which the likelihood of the value coming from background and signal distribution equals (Figure S4).

For purpose of an unbiased evaluation of model performance, the data were divided into training (265560 bins), validation (parameter selection, 84976 bins) and hold-out testing set (183859 bins). To minimize dependency between each set, instead of randomly assigning bins we first divided genome to 50 approximately equal-sized slices. 25 slices were assigned to training set. The other 25 slices were further divided into validation set and testing set. Training set was used for learning models; validation set was for selecting tuning parameters like regularization parameter in structure learning step; and test set was used only for evaluating model performance.

### Model coherence score calculation

Probability distribution was evaluated by coherence score, which was calculated as exponential of mean log-likelihood of each chromatin code in test data. Thus higher coherence score indicates better model of data. For maximum entropy models, we first need to estimate the normalization constant, or partition function Z. Z was estimated by one divided by proportion of all-zero pattern in samples drawn from the model. As all-zero pattern is the most common pattern, this estimator can provide a reliable estimation of Z. 1,000,000 samples were drawn with Gibbs sampling for maximum entropy model (one sample per iteration of sequential Gibbs sampling sweep). The probability for each pattern can then be easily calculated. For independent Bernoulli model, probability can be directly calculated by the product of empirical marginal probability of each chromatin factor.

### Context based prediction of chromatin profiles and evaluation

Given ChIP profiles for a subset of chromatin factors, the unknown chromatin factor profiles can be estimated by conditional probability based on their interactions with profiled factors. The conditional probability formula for each chromatin factor given the others can be easily derived given the maximum entropy model:
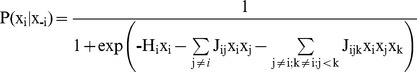
(6)


To evaluate the accuracy of prediction with S2-DRSC cell model, we calculated the conditional probability of each chromatin factor at each genomic bin based on profiles of remaining chromatin factor in test set data. The conditional probabilities for each bin were then compared with observation, and we calculated Area under Receiver Operating Characteristic curve (AUC) as the evaluation metric. For cross-cell-type prediction evaluation, we similarly trained 3^rd^-order maximum entropy models on the data for the consensus set of 47 chromatin factors measured for S2-DRSC or DmBG3 cell. Intra- and cross- cell type predictions were computed and evaluated on the test set data as described above.

### Prediction of actively transcribed regions and evaluation

For prediction of actively transcribed regions, we trained maximum entropy models using both 73 chromatin factors and RNA-seq data for S2-DRSC cells from [46]. RNA-seq data were processed by the same thresholding algorithm as for processing ChIP data with the difference that the discretization threshold is decided for per-gene Reads per kilo base per million (RPKM) measures.

## Supporting Information

Figure S1 **Comparison of pairwise interaction energy scores with Bayesian network bootstrap scores and correlations.** (A) Pairs with both strong positive and negative pairwise interaction energy scores tend to have higher Bayesian network bootstrap scores. (B) Pairwise correlations show only weak correlation with interaction energy scores.
(EPS)

Figure S2 **Comparison of pairwise interaction Maximum entropy model interaction energy scores using data preprocessed with different bin width.** Pairwise interaction model with regularization trained with data preprocessed with 200 bp were compared with model trained with the same procedure but using 500 bp bins in preprocessing. Model interaction energy scores are not sensitive to the bin width difference in data preprocessing.
(EPS)

Figure S3
**Comparison between S2-DRSC cell and DmBG3-c2 cell pairwise interaction maximum entropy models with regularization.** (A) and (B) show comparison of maximum entropy model parameters. In (A) upper right triangle and lower left triangle show interaction energy score 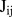
s of S2 cell model and BG3 cell respectively. In (B), green dots shows chromatin factor self-energy score 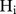
s, while black dots show interaction energy scores 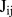
s.(EPS)

Figure S4 **Examples of chromatin factor standardized binned ChIP signal distribution and discretization threshold.** (A–F) Solid line represents the overall standardized binned ChIP signal distribution for the chromatin factor, dashed line represents estimated background and signal distribution. Vertical solid line represents the optimal discretization threshold determined by our thresholding algorithm.
(TIF)

Table S1 **Interaction energy scores from the regularized pairwise interaction maximum entropy model of S2-DRSC cell based on modENCODE datasets.**
(TXT)

Table S2 **Curated experimentally supported direct positive interactions for the evaluation of pairwise interaction prediction.**
(TXT)

Table S3 **Top triplet interaction energy scores from the 3rd order interaction maximum entropy model of S2-DRSC cell based on modENCODE datasets.**
(TXT)

Table S4 **Area under ROC (AUC) scores for evaluation of predicting test set chromatin profiles by the S2-DRSC cell 3rd-order maximum entropy model.**
(TXT)

Text S1 **Testing multivariate normality of the chromatin profile data.**
(DOCX)
