# Supplementary material for: Global Quantitative Modeling of Chromatin Factor Interactions
Source: PLoS Comput Biol. 2014 Mar 27;10(3):e1003525. doi: 10.1371/journal.pcbi.1003525 (PMC3967939; doi:10.1371/journal.pcbi.1003525)
Supplement: Text S1 — Testing multivariate normality of the chromatin profile data. (DOCX) [file pcbi.1003525.s009.docx]

**Supplementary Text**

**Testing for multivariate normality of chromatin profile data**

To decide whether the joint distribution of chromatin factor binding signals from ChIP-chip experiments processed by MAT algorithm (log Experiment/Control) is multivariate Gaussian distribution, we tested the data for multivariate normality.

With Shapiro-Wilk test for multivariate normality on 5000 samples, the distribution is significantly different from multivariate Gaussian:

W = 0.7949, p-value < 2.2E-16

We also tried to force distribution of each chromatin factor to be Gaussian by nonparametric transformation. Even after transformation, the joint distribution is still significantly different from multivariate Gaussian.

With nonparametric transformation:

W = 0.9305, p-value < 2.2E-16
